# Minimal self-models and the free energy principle

**DOI:** 10.3389/fnhum.2013.00547

**Published:** 2013-09-12

**Authors:** Jakub Limanowski, Felix Blankenburg

**Affiliations:** ^1^Berlin School of Mind and Brain, Humboldt-Universität zu BerlinBerlin, Germany; ^2^Dahlem Institute for Neuroimaging of Emotion, Freie Universität BerlinBerlin, Germany; ^3^Center for Adaptive Rationality (ARC), Max Planck Institute for Human DevelopmentBerlin, Germany

**Keywords:** free energy principle, predictive coding, active inference, self, minimal phenomenal selfhood, ownership, agency, self-model

## Abstract

The term “minimal phenomenal selfhood” (MPS) describes the basic, pre-reflective experience of being a self (Blanke and Metzinger, 2009). Theoretical accounts of the minimal self have long recognized the importance and the ambivalence of the body as both part of the physical world, and the enabling condition for being in this world (Gallagher, 2005a; Grafton, 2009). A recent account of MPS (Metzinger, 2004a) centers on the consideration that minimal selfhood emerges as the result of basic self-modeling mechanisms, thereby being founded on pre-reflective bodily processes. The free energy principle (FEP; Friston, 2010) is a novel unified theory of cortical function built upon the imperative that self-organizing systems entail hierarchical generative models of the causes of their sensory input, which are optimized by minimizing free energy as an approximation of the log-likelihood of the model. The implementation of the FEP via predictive coding mechanisms and in particular the active inference principle emphasizes the role of embodiment for predictive self-modeling, which has been appreciated in recent publications. In this review, we provide an overview of these conceptions and illustrate thereby the potential power of the FEP in explaining the mechanisms underlying minimal selfhood and its key constituents, multisensory integration, interoception, agency, perspective, and the experience of mineness. We conclude that the conceptualization of MPS can be well mapped onto a hierarchical generative model furnished by the FEP and may constitute the basis for higher-level, cognitive forms of self-referral, as well as the understanding of other minds.

## Introduction

What lets an organism be a self? Throughout philosophical attempts to understand the enabling conditions of minimal self-awareness (Zahavi, 1999), or *minimal phenomenal selfhood* (MPS)^1^ (Blanke and Metzinger, 2009), the special status of the body among all other physical things has long been apparent (Merleau-Ponty, 1962; Bermúdez et al., 1998; Anderson and Perlis, 2005; Legrand, 2006; Blanke, 2012). Recently, the role of the human body for cognition has been re-emphasized in the field of embodied cognition (Varela et al., 1994; Clark, 1999; Gallagher, 2005a; Grafton, 2009; Gallese and Sinigaglia, 2011). The body lets us interact with the world via perception and action (Legrand, 2006; Friston, 2011; Farmer and Tsakiris, 2012), leading to a whole new form of intelligence that is different from, for example, mere computation (Frith, 2007; Grafton, 2009). One’s everyday experience is enabled and structured through a body that is “always there” (James, 1890), and hence the body—*my* body—is not just part of the physical world, but also the “vehicle” that enables being a self in this world (Merleau-Ponty, 1962; Varela et al., 1994; Gallagher, 2005a). Minimal, or pre-reflective selfhood emerges from this experience of a unified, situated living body as a “sensorimotor unity anchored to its world” (Bermúdez et al., 1998; Anderson and Perlis, 2005; Gallagher, 2005a; Legrand, 2006; Hohwy, 2010; Blanke, 2012; Apps and Tsakiris, 2013).

In this review, we will particularly consider an account of the mechanisms giving rise to minimal selfhood that has recently been proposed by Metzinger (2003, 2004a,b, 2005). Central to the theory is the premise that minimal selfhood emerges as the result of pre-reflective self-modeling, i.e., through an organism’s model of the world that is phenomenologically centered onto the self. Thereby, Metzinger’s account builds on the proposition that the brain is a representational system that needs to interpret the world (Gallese and Metzinger, 2003), and thus constructs and simulates a model in order to reduce ambiguity originating from the external world (Metzinger, 2005). For this system-model to be successful, i.e., of adaptive value, “the self needs to be embedded into the causal network of the physical world” (Knoblich et al., 2003; Metzinger, 2004a, 2005). The model thus also has to include as part of itself the physical body—“the part of the simulation that represents the system itself” (Edelman, 2008, p. 419). Metzinger (2004a) emphasizes that this self-representation of the system is special in that it (i.e., the body) is the only representational structure that constantly generates and receives internal input via its different intero- and proprioceptive systems. Notably, a resulting structural property of the system-model is the spatiotemporal centeredness of the model onto a coherent phenomenal subject, described by Metzinger with the term *perspectivalness* (Metzinger, 2004a, 2005; Blanke and Metzinger, 2009). Throughout this review, we will return to this, and propose to understand it as an instance of “perspective taking”, whereby the brain assigns the subjective, first-person perspective (1PP) to its self-model.

Following their emphasis of self-modeling mechanisms for minimal selfhood, Metzinger and colleagues (Knoblich et al., 2003) have argued that an analysis of selfhood should focus on the underlying *functional* properties of the system, i.e., the brain. In this review, we will examine one promising candidate brain theory for this analysis: over the last years, a general theoretical account of cortical function based on the “free energy principle” (FEP) has been put forth by Friston (Friston et al., 2006; Friston, 2009, 2010; Clark, 2013), based on the conclusive assumption that the brain entails hierarchical dynamical models to predict the causes of its sensory data (Hohwy, 2007; Frith, 2007; Friston and Kiebel, 2009; Bubic et al., 2010).

The key premise of the FEP is that self-organizing organisms have to resist the natural tendency to disorder that is implied by the second law of thermodynamics, i.e., they have to “maintain their states and form in the face of a constantly changing environment” (Friston, 2010). Organisms do so by avoiding *surprise* associated with their sensory states (Friston et al., 2011, 2012; Friston, 2012a,b), which in turn will result in a (desired) state where the world is highly predictable. The FEP proposes that the brain infers the hidden causes of the environment via the inversion of hierarchical generative models that predict their sensory consequences (Friston, 2010; Bastos et al., 2012), with higher levels encoding increasingly abstract and information-integrating conceptions of the world (Fotopoulou, 2012; Clark, 2013). Importantly, as biological organisms are embodied in the environment, the “world-model” of a self-organizing system also has to include the sensory apparatus (the body) of the organism (Friston, 2012b; Friston et al., 2012; Clark, 2013). In agreement with the Good Regulator theorem (Conant and Ashby, 1970; Edelman, 2008; Friston et al., 2012), which states that every good regulator of a system will ultimately become a model of that system, the FEP thus proposes as a consequence of hierarchical predictive modeling that “I model myself as existing” (Friston, 2011, 2013b). We will later highlight that this conforms nicely to accounts of minimal selfhood, whereby the self is perceived as a result of dynamic self-modeling mechanisms (Metzinger, 2004a; Hohwy, 2007).

Conceptually, the FEP is based on the evaluation of the improbability of some sensory data under a hierarchical generative model, where the (model-conditional) improbability of the data is commonly referred to as *surprise* (Friston et al., 2006; Friston, 2010, 2011). The theory builds on *free energy* as an information-theoretical quantity on the upper bound of surprise that can be formally assessed (Friston et al., 2006, 2012; Friston, 2010, 2011). By minimizing free energy within a model, biological agents thus always also minimize surprise. In principle, this can be done in two ways: By changing the *predictions of the model* by means of perception, or by changing *what is predicted* by selectively sampling those sensations that confirm the model’s predictions by means of action (a “systematic bias in input sampling”, Verschure et al., 2003; Friston, 2011).

Minimizing surprise associated with sensory data by the inversion of the hierarchical generative model (and the dynamic optimization of its parameters) has been established as *predictive coding* (Srinivasan et al., 1982; Mumford, 1992; Rao and Ballard, 1999; Friston, 2005a; Friston and Stephan, 2007; Kilner et al., 2007; Friston and Kiebel, 2009). Thereby, the predictive coding scheme infers the hidden causes of its sensory input by minimizing the difference between the predictions about sensory data and the actual sensory data at any level of the model’s hierarchy, which is encoded by the *prediction error* (Friston and Kiebel, 2009; Bubic et al., 2010; Friston, 2010; Brown and Brüne, 2012; Friston, 2012a). Thus the *feedforward* signal is not the sensory information *per se*, but the associated prediction error that is passed up the hierarchy (Hohwy, 2012; Clark, 2013), while the generative model’s predictions are the *feedback* signal (Friston, 2010; Bastos et al., 2012; Edwards et al., 2012). The second form of prediction error minimization via interaction with the environment is described under the *active inference* principle (Friston, 2012a, 2013a). Reminiscent of “affordances”, Gibson’s (1977) famous description of the fact that the environment is “co-perceived” depending on the perceiver’s bodily endowment, active inference thus emphasizes the bi-directional role of embodiment such that “not only does the agent embody the environment but the environment embodies the agent” (Friston, 2011). Interestingly, the computational assumptions of predictive coding are surprisingly well reflected by neuroanatomical organization of the cortex (Bastos et al., 2012; Friston, 2012a), suggesting that neuronal populations indeed encode probabilities, i.e., uncertainty (Clark, 2013). In sum, predictive coding and active inference are neurobiologically plausible, “action-oriented” (Bastos et al., 2012; Clark, 2013) implementations of free energy minimization (Friston, 2011; Bastos et al., 2012; Friston, 2012a; Clark, 2013).

In this review, we summarize recently formulated free energy accounts of key aspects of minimal selfhood: multisensory integration, interoception, agency, ownership or “mineness” of experience, the perspectivity of self-models and models of other selves. Common to these FEP applications is the focus on “self modeling” (Friston, 2012a). We hence consider these approaches in the light of the proposal that the minimal self is the result of an ongoing predictive process within a generative model that is centered onto the organism (Metzinger, 2004a; Hohwy, 2007; Friston, 2011).

## Aspects of the minimal self in the free energy framework

A number of publications have recently put forward the idea that (minimal) selfhood is based on the neurobiological implementation of hierarchical generative models in the brain (Hohwy, 2007, 2010; Seth et al., 2011; Fotopoulou, 2012; Friston, 2012a,b; Apps and Tsakiris, 2013; Clark, 2013). In one sentence, these accounts propose to “understand the elusive sense of minimal self in terms of having internal models that successfully predict or match the sensory consequences of our own movement, our intentions in action, and our sensory input” (Hohwy, 2007). In accordance with Friston (2011, 2012b, 2013b), who has already emphasized the fundamental, bi-directional role of embodiment in the FEP, these accounts also embrace the body as a central part of the self-model. The aspects of the minimal self that these approaches formalize in the FEP all follow as consequences from this embodied self-modeling (Metzinger, 2004a; Hohwy, 2007; Friston, 2011): The body predicts and integrates multisensory information in a way that no other physical object does (Hohwy, 2007, 2010; Apps and Tsakiris, 2013), it is the only source of internally generated input (Seth et al., 2011; Critchley and Seth, 2012), it is crucial for interaction with the environment and a sense of agency (Kilner et al., 2007; Frith, 2007; Friston et al., 2011). From the phenomenological and spatiotemporal centeredness of experience onto the body (Friston, 2011) emerges the 1PP, and ultimately, the “mineness” of experience (Hohwy, 2007; Apps and Tsakiris, 2013).

### Multisensory integration

A very important implication of the free energy framework is that sensory information is processed probabilistically, and thus it follows that the representation of the self is also probabilistic (Friston, 2011). This conceptualization fits comfortably with Metzinger’s (2004b) theory, where the content of the self-model is probabilistic, i.e., it is “simply the best hypothesis about the current state of the system, given all constraints and information resources currently available” (see also Hohwy, 2010; Clark, 2013; Friston, 2013b). However, sensory information is not *per se* specific to the self, which implies that there must be additional levels of information processing in which information is related to the self (Apps and Tsakiris, 2013).

Previous accounts of bodily self-awareness, inspired by work on illusions of body ownership and related paradigms, have emphasized the role of multimodal, hierarchical cortical networks in processing self-related information (Hohwy, 2007, 2010; Tsakiris, 2010; Petkova et al., 2011a; Blanke, 2012). In a recent paper, Apps and Tsakiris (2013) propose that hierarchical prediction error minimization can explain processes of self-recognition and self-representation: for the processing of information relating to the self, free energy minimization happens via the integration of various streams of surprise from unimodal sensory information in hierarchically higher multimodal areas, where information from any system can be used to “explain away” surprise in any other system (Hohwy, 2010; Apps and Tsakiris, 2013; Clark, 2013). This corresponds to the basic claim of predictive coding about crossmodal information processing, according to which hierarchically higher levels form amodal concepts that generate multimodal predictions and prediction errors (Friston, 2012a). Following this logic, higher-level multisensory areas must predict input in multiple sensory modalities, which according to Apps and Tsakiris (2013) implies “a high level representation (of self) that elaborates descending predictions to multiple unimodal systems” (see also Clark, 2013; Friston, 2013b). This self-model can thus be seen as the most accurate, immediately available explanation of the bottom-up surprise from incoming multisensory information (Apps and Tsakiris, 2013; thereby the model need not be “true”, just a *sufficient* explanation of the sensory input, Schwabe and Blanke, 2008; Hohwy and Paton, 2010; Hohwy, 2012). The predictive coding account suggests that, at the hierarchically highest level, such a self-model will encode, as model evidence, the evidence for the existence of the agent in the present form (Hohwy, 2010; Friston, 2011).

A particularly intriguing example of how self-representation is constructed in a probabilistic way is the rubber hand illusion (RHI; Botvinick and Cohen, 1998): observing a dummy hand being touched, while receiving synchronous tactile stimulation at the anatomically congruent location of one’s real, hidden hand typically leads to an illusory experience of feeling the touch on the dummy hand (Botvinick and Cohen, 1998; Ehrsson et al., 2004, 2005; Makin et al., 2008). This usually results in a self-attribution, or “incorporation” (Holmes and Spence, 2004) of the fake hand as a part of one’s own body (Tsakiris and Haggard, 2005; Hohwy and Paton, 2010; Tsakiris, 2010; Petkova et al., 2011a). A number of behavioral measures such as a fear response to the dummy hand being threatened (Armel and Ramachandran, 2003; Ehrsson et al., 2007), or the mislocalization of one’s real hand towards the location where the dummy hand is seen (Botvinick and Cohen, 1998; Tsakiris and Haggard, 2005), suggest that the brain indeed seems to treat the dummy hand as part of the body as a result of the multisensory stimulation (see Tsakiris, 2010, or Blanke, 2012, for detailed reviews). Using virtual reality techniques, the RHI paradigm has been extended to induce an illusory self-identification with a whole dummy body located at a different position in space (Ehrsson, 2007; Lenggenhager et al., 2007). In those cases, participants exhibited a bias in judging their own spatial location towards the location where the dummy body was positioned in space, just as the mislocalization of the own hand during the RHI (see Blanke, 2012, for a review). These findings thus impressively demonstrate that perceived self-location can be manipulated with appropriate stimulation.

Generally, illusory percepts are well explained as a result of Bayes-optimal inference, i.e., arising from an interpretation of ambiguous sensory input under strong prior hypotheses (Friston, 2005b; Brown and Friston, 2012; Apps and Tsakiris, 2013; Clark, 2013). Correspondingly, a combination of bottom-up input and modulatory top-down factors has been suggested to drive illusory ownership of body parts as experienced during the RHI (de Vignemont et al., 2005; Tsakiris and Haggard, 2005; de Preester and Tsakiris, 2009; Hohwy and Paton, 2010; Tsakiris, 2010). While congruent multisensory input seems crucial for the RHI (Botvinick and Cohen, 1998; Armel and Ramachandran, 2003; Ehrsson et al., 2004, 2005; Hohwy and Paton, 2010; Petkova et al., 2011a), there have been strong arguments for top-down “body representations” that define which objects (namely, only anatomically plausible hand-shaped objects, see e.g., Tsakiris and Haggard, 2005) can be incorporated during the RHI (de Vignemont et al., 2005; IJsselsteijn et al., 2006; Costantini and Haggard, 2007; Tsakiris et al., 2007; de Preester and Tsakiris, 2009). However, various inconsistent definitions of body representations may have lead to some confusion and thus prevented the emergence of a unifying theoretical account (de Vignemont, 2007; Longo et al., 2008; Apps and Tsakiris, 2013).

As a solution to this problem, several authors have endorsed a predictive coding approach (Hohwy, 2007, 2010; Apps and Tsakiris, 2013). Consider that, under normal circumstances, observed touch on our skin is accompanied by a corresponding, temporally congruent tactile sensation—in predictive coding terms, the underlying generative model of our physical self predicts a somatosensory sensation when touch is about to occur on the body, because associations between events that have a high probability of predicting events in another system lead to the formation of beliefs, or priors on a hierarchically higher level (Apps and Tsakiris, 2013). Note that it are not *per se* the associations between different kinds of sensory input that are of importance here, but the parallel predictions of the generative model. Among all physical objects in the world, it is only our body that will evoke (i.e., predicts) this kind of multisensory sensation—congruence of multisensory input has (not surprisingly) been called “self-specifying” (Botvinick, 2004) and has been ascribed a crucial role in self-representation (Botvinick and Cohen, 1998; Armel and Ramachandran, 2003; Ehrsson et al., 2005; Hohwy and Paton, 2010). Following this logic, during the RHI, surprise^2^ or prediction error is evoked by the simultaneous occurrence of observed touch on an external object (the dummy hand) together with a somatosensory sensation, because such congruence is not predicted by the brain’s initial generative model.

The predictive coding account suggests that, as stimuli can usually be caused “in an infinite number of ways” (Brown and Friston, 2012), there are several competing explanations of the sensory input between which the brain needs to decide. In the case of the RHI, these are coded by the probabilities of the actual hand, or the dummy hand being “me” (Apps and Tsakiris, 2013). One explanation, or model, of the sensory input is that vision and touch occur at different locations (the “true” model, Hohwy, 2010). However, during the RHI, spatially distributed observed and felt touch are “bound together” by causal inference (Hohwy, 2012): this “false” model (that observed and felt touch occur at the same location, namely, one’s own hand) is selected because it more successfully explains the incoming prediction error in favor of a unified self (see also Schwabe and Blanke, 2008; Hohwy, 2010; Hohwy and Paton, 2010). This is a crucial point, because predictive coding is a “winner takes all” strategy (Hohwy, 2007, 2010): there is always one model that has the lowest amount of free energy (the highest model evidence) among all possible models of the sensory input (Friston et al., 2012; Apps and Tsakiris, 2013; Clark, 2013), and this model is selected as the explanation for the world. This model does not have to be “true”, just a better explanation of the sensory input than competing models (Friston et al., 2012). As minimizing surprise is the same as maximizing model-evidence (where model-evidence is evidence for the agent’s existence), the agent, or self, in its present form will cease to exist if another model has to be chosen as a better explanation of sensory input (Hohwy, 2010; Friston, 2011): “I” (i.e., the embodied model of the world) will only exist “iff (sic) I am a veridical model of my environment” (Friston, 2011).

Applied to the RHI example, this means that if prediction error could not be explained away in this way, the system might have to dismiss its current self-model in favor of a better explanation of the input—which would result in the representation of a “disunified self” (Hohwy, 2010). The FEP states that, if prediction error can be explained away at lower levels, there is no need to adjust higher-level representations (Friston, 2012a). Apps and Tsakiris (2013) propose that, as the prediction error is passed up the hierarchy during the RHI, it can be explained away at multimodal cortical nodes. Thereby “explaining away” means an updating of the generative model’s predictions about the physical features of the self to minimize the overall level of surprise in the system. This results in a different posterior probabilistic representation of certain *features* of the self (Hohwy and Paton, 2010; Apps and Tsakiris, 2013), however, without any necessity to change the actual generative self-*model* (Hohwy, 2010). Specifically, the dummy hand is now probabilistically more likely to be represented as part of one’s body, which in turn is accompanied by a decrease in the probability that one’s actual hand will be represented as “self”. This manifests as a self-attribution of the dummy hand, and a partial rejection of the real limb (de Preester and Tsakiris, 2009; Tsakiris, 2010).

Indeed, there is compelling experimental evidence in support of such a probabilistic integration process underlying the RHI. For example, the mislocalization of one’s real hand towards the location of the dummy hand is never absolute, but relative; participants usually judge the location of their hand several centimeters closer to the dummy, but not at the same location (Tsakiris and Haggard, 2005). Lloyd (2007) showed that the RHI gradually decreases with increasing distance between the own and the dummy hand. Furthermore, a drop in skin temperature of the stimulated real hand was found to accompany the RHI (Moseley et al., 2008), which has been interpreted as evidence for top-down regulations of autonomic control and interoceptive prediction error minimization during the RHI (Moseley et al., 2008; Seth et al., 2011; Suzuki et al., 2013). Also, after the illusion, the dummy hand is frequently perceived as more similar to one’s real hand (Longo et al., 2009). These findings suggest that in fact, explaining away prediction error from ambiguous multisensory stimulation may lead to changes in the encoded features of the self (Hohwy and Paton, 2010).

The idea of a probabilistic self-representation in the brain benefits from the fact that the free energy account is relatively unconstrained and thus not as heavily dependent on conceptual assumptions as other theories (Hohwy, 2007, 2010; Friston, 2008; Friston and Kiebel, 2009; Friston et al., 2012). Thus the FEP does not need to treat information relating to the self as a distinct class of information (Apps and Tsakiris, 2013), because it is concerned with information flow and system structure. For example, the matching of sensory predictions based on corollary discharge with actual sensory input has been previously proposed as a basis for self-awareness (see Gallagher, 2000; Brown et al., 2013). In the free energy account, however, self-awareness is not restricted to the integration of sensorimotor efference and re-afference. Rather, *any* type of sensory information can be integrated within a multimodal, abstract representation of the self, and explain away surprise in another system (Apps and Tsakiris, 2013). The RHI example demonstrates that, as claimed by the FEP (Friston, 2012a), if prediction error can be explained away in the periphery (e.g., adjusting the encoded location of one’s real hand), there is no need to adjust higher-level representations (the unified self-model). The FEP is thus a parsimonious, and hence inherently flexible, formal description of how multisensory information integration underpins minimal forms of self-awareness (Hohwy, 2010; Blanke, 2012).

### Interoception

A special case of information that the self-model receives is input from interoceptive senses: within the world-model, the (own) body is special among all physical objects in that it constantly receives a “background buzz” of somatosensory input, including input from somato-visceral and mechanoreceptors, and higher-level feeling states (Metzinger, 2004a, 2005; see Friston, 2011). Acknowledging the importance of interoception, recent work by Seth (Critchley and Seth, 2012; Seth et al., 2011; Suzuki et al., 2013) has promoted interoceptive prediction error minimization as a mechanism for self-representation. Specifically, Seth et al. provide a predictive coding account of “presence”, where presence means the subjective experience of being in the here and now (see Metzinger, 2004a). Presence is hence a structural property of conscious experience (Seth, 2009) that is transparent in the sense that Metzinger (2003) uses the term (Seth et al., 2011). According to Seth et al. (2011), interoceptive predictions arise from autonomic control signals and sensory inputs evoked by motor control signals. The generative model of the causes of interoceptive input gives rise to “interoceptive self-representations” and “emotional feeling states” (Suzuki et al., 2013). Presence results as the successful suppression of the associated prediction error (Seth et al., 2011), more specifically, “self-consciousness is grounded on the feeling states that emerge from interaction of interoceptive predictions and prediction errors” (Critchley and Seth, 2012). The emphasis on subjective feeling states (Critchley et al., 2004; Seth et al., 2011) as a key component of interoceptive predictive coding links this account to emotion frameworks like the somatic marker hypothesis (Damasio, 1999; Bechara et al., 2000).

Half a century ago, Schachter and Singer (1962) showed that people seek explanations for their bodily sensations after having become aware of them. Reversing this argument, Pennebaker and Skelton (1981) showed that the perception of bodily sensations depended on the hypotheses held by the participants, and was thus not different from the processing of any other ambiguous information. More recently, Moseley et al. (2008) found that the RHI led to a cooling of participants’ real hand (and only the hand affected by the illusion), and concluded that there is a causal link between self-awareness and homeostatic regulation, where bodily self-awareness regulates physiological processing in a top-down manner. In accordance with these results, the FEP indicates that interoceptive predictions are “one—among many—of multimodal predictions that emanate from high-level hypotheses about our embodied state.” (Friston, 2013b; Suzuki et al., 2013). Interestingly, as we will see later (see *Modeling Others*), these predictions can also be used to model others’ internal states (Bernhardt and Singer, 2012). In sum, although predictive coding accounts of interoception still need detailed work, the corresponding emphasis of interoceptive signals by predictive coding (Seth et al., 2011) and philosophical (Metzinger, 2004a) accounts of the self promises many insightful studies to come.

### Action and agency

Agency as a “sense of initiative” (Edelman, 2008) has been emphasized as a key component of MPS (Gallagher, 2000; Metzinger, 2004a; Frith, 2007). Distinguishing between self-initiated actions and actions of other organisms is crucial for being a self. The importance of the motor system in the brain’s ontology (interpretation) of the world (Gallese and Metzinger, 2003) has been promoted by forward models of agency based on corollary discharge (Blakemore et al., 2002; Gallagher, 2005a; Frith, 2012), which have also been applied to describe disturbances of agency resulting from a failure of these mechanisms (Gallagher, 2000). Advancing on these accounts, action and the phenomenology of agency have both been accounted for in terms of hierarchical generative models (Hohwy, 2007).

The active inference principle is of central importance in the FEP (Friston and Stephan, 2007; Hohwy, 2007, 2010; Kilner et al., 2007; Brown et al., 2013; Friston, 2013a): action changes the sensory input of an organism so that it better corresponds to the current generative model, without having to revise the model parameters (Friston and Stephan, 2007; Hohwy, 2010). This validation of the current generative system-model is a confirmation of the agent’s existence (Friston, 2011). However, for active inference to be feasible, the agent has to be able to predict which actions will lead to a better confirmation of its predictions. Friston (2012b) thus states that “implicit in a model of sampling is a representation or *sense of agency”*, since the effects of selective sampling of sensations as through active inference have to be known—modeled—as well. Thus, by selectively sampling sensations so that they confirm the model’s predictions, action is a form of “reality testing” (Hohwy, 2007). For instance, consider that the induction of illusory limb or body ownership via multisensory stimulation (like in the RHI) only works because this kind of active inference is suppressed.^3^ If allowed, participants would probably instantaneously move their hand to *test* whether the rubber hand moves as well. The illusion will be immediately abolished once participants see that the rubber hand does not move according to their intentions (IJsselsteijn et al., 2006; Slater et al., 2009; Maselli and Slater, 2013), because now there is a clear mismatch between predicted and actual sensory outcome, which cannot be explained away.

It is noteworthy that failures in basic inference mechanisms are a likely cause of many symptoms connected to a disturbed sense of agency (Gallagher, 2000; Frith, 2007). As stated by the FEP, probabilistic inference under uncertainty underlies all perception, and it thus seems reasonable to explain abnormal experiences in the same framework (Fletcher and Frith, 2008; Hohwy, 2013). Predictive coding schemes and Bayesian inference have been successfully applied to explain symptoms like delusion formation (Fletcher and Frith, 2008; Hohwy, 2013) or failures in sensory attenuation occurring in schizophrenia (Brown et al., 2013), hysteria or functional symptoms (Edwards et al., 2012), out-of-body experiences (Schwabe and Blanke, 2008), and depersonalization (Seth et al., 2011). In many of these cases, basic mechanisms of active inference fail (Brown et al., 2013), but it is not yet clear whether these symptoms can be explained by failures at low levels alone, or rather by a failure of mechanisms across the hierarchy (Fletcher and Frith, 2008). For instance, a noisy prediction error signal has been suggested as the cause for positive symptoms in schizophrenia (Fletcher and Frith, 2008), while delusions are seen as the result of false inference “at a conceptual level” (Brown et al., 2013), which may be characterized by a “lack of independent sources of evidence for reality testing” (Hohwy, 2013).

In conclusion, action and agency are of fundamental importance for the experience of normal minimal selfhood. However, although a sense of agency (Gallagher, 2000) is sufficient for MPS, it may not be the most basal constituent (Blanke and Metzinger, 2009). What matters is that I experience the action as *mine* (Gallagher, 2000), which brings us to the most important aspect of the generative self-model: the experience of “mineness” (Hohwy, 2007).

### Mineness

The phenomenal experience of “mineness” is a key property of MPS (Metzinger, 2004a). The idea that the living body is experienced as mine (“owned”) can be traced back to early phenomenologists like Merleau-Ponty or Husserl (see Gallagher, 1986, 2009). It has been claimed that this “self-ownership” (Gallagher, 2000) is the most fundamental sense of phenomenal selfhood (Aspell et al., 2009; Blanke and Metzinger, 2009). Similarly, Hohwy (2007) equates experienced mineness of actions and perceptions with the experience of a minimal self.

In Hohwy’s (2007) FEP account of the self, mineness is a general phenomenon, resulting from successful predictions of actions and perceptions. It is hereby important to keep in mind that prediction is more than mere anticipation (Hohwy, 2007; Bubic et al., 2010), but describes predictive *modeling* as a fundamental principle of the brain, and that what is informative in predictive coding is the prediction *error*. Following Hohwy’s (2007) logic, phenomenal selfhood thus arises as a consequence of successfully having predicted incoming sensory input across the hierarchy of the self-model. Within predictive coding, prediction error is not explained away post-hoc, but constantly, and across all levels of the model (Friston, 2012a). Thus mineness is always *implicit* in the flow of information within the hierarchical generative self-model, and can correspondingly be experienced for actions and perceptions in the same way (note how once again the FEP is simple in its assumptions). Crucially, this means that the minimal self is the result of an ongoing, dynamic process, not a static representation. In this account, mineness is thus situated in a spatio*temporal* reference frame (see Metzinger, 2004a; Hohwy, 2007), where prediction introduces the temporal component of “being already familiar” with the predicted input (Hohwy, 2007; see Kiebel et al., 2008; Bubic et al., 2010).

Perhaps a good example for this construction of temporally extended phenomenal experience from predictive processes is the classical concept of a *body schema* (Head and Holmes, 1911–1912; Merleau-Ponty, 1962). The body schema describes the dynamic organization of sensorimotor processes subserving motor and postural functions in a form of “embodied memory” that ultimately presents the body for action (Gallagher, 2009). These processes are pre-reflective, operating “below the level of self-referential intentionality” (Gallagher and Cole, 1995), and thus the body schema is not a static representation (Gallagher, 2005a). But note that the body schema defines the range of possible actions that my body can perform, while being “charged” with what has happened before (see Gallagher, 2009, for a nice review). In the hierarchical generative self-model, the body schema might thus be pictured as encoded by a structure of predictions (e.g., of self-location and proprioception).

In conclusion, the following picture seems to emerge from the reviewed literature: the FEP is capable of describing the functional regularities of the brain’s “ontology” (Gallese and Metzinger, 2003), such as the prediction and integration of intero- and exteroceptive signals (Hohwy, 2010; Seth et al., 2011; Apps and Tsakiris, 2013), the importance of action and agency (Gallagher, 2000; Hohwy, 2007; Friston, 2012a), and the mineness of experience (Hohwy, 2007, 2010). In agreement with the Good Regulator theorem (Conant and Ashby, 1970; Edelman, 2008; Friston et al., 2012), which states that every good regulator of a system will ultimately become a model of that system, both the FEP and the philosophical account of minimal selfhood agree that the agent *is* the current embodied model of the world (Metzinger, 2004a; Hohwy, 2007; Friston, 2011).

### The perspectivity of the self-model

In accordance with the FEP, the phenomenal self-model (PSM) theory views selves as processes, not objects. Accordingly, the self is perceived *because* systems with a PSM constantly assume, or model, their own existence as a coherent entity (Metzinger, 2004a; Blanke and Metzinger, 2009). However, to assume that there is a perceiver is a fallacy (“no such things as selves exist in the world”, Metzinger, 2005). Rather, a conscious self is a result of the system’s identification with its self-model (“you *are* the content of your PSM”, Metzinger, 2005).

This self-identification is possible because the “attentional unavailability of earlier processing stages in the brain for introspection” (Metzinger, 2003, 2005) leads to a gradually increasing *transparency* of higher-level phenomenal states. Transparency thus describes the fact that only the contents of phenomenal states, not their underlying mechanisms, are introspectively accessible to the subject of experience (Metzinger, 2003, 2004a). Interestingly, it has been proposed that the cognitive impenetrability of predictive coding mechanisms can be explained by the fact that hierarchically higher levels predict on longer timescales, and more abstractly than lower levels (Hohwy, 2007, 2010; Kiebel et al., 2008). Failures in these mechanisms may result in severe symptoms that seem to be related to a loss of global experiential selfhood, as demonstrated by certain disorders of “presence” such as depersonalization disorder (Seth et al., 2011). These phenomena might also be described by a loss of transparency (“if … the self-model of a conscious system would become fully opaque, then the phenomenal target property of experiential “selfhood” would disappear”, Metzinger, 2004b).

Thus, the crucial implication of transparency is that the PSM “cannot be recognized as a model by the system using it” (Metzinger, 2004a), which greatly reduces computational load within the system by efficiently avoiding an infinite regression that would otherwise arise from the logical structure of self-modeling (Metzinger, 2004a, 2005): “I can never conceive of what it is like to be me, because that would require the number of recursions I can physically entertain, plus one” (Friston et al., 2012). Similarly, the FEP states that systems operating with a self-model will have an advantage because “a unified self-model is what best allows computation of the system’s current state such that action can be undertaken” (Hohwy, 2010; see Friston et al., 2012, for a discussion).

Note how, by the transparent spatiotemporal centeredness of the model onto the self (Metzinger, 2003, 2004a; see also Hohwy, 2007; Friston, 2011, 2012b), the model takes on a 1PP (Vogeley and Fink, 2003). However, the centeredness of the model is *phenomenal*, and not just (but also) geometrical (a temporal centering on the subject happens through successful prediction, see previous section). This is well reflected by Blanke and Metzinger (2009), who distinguish between the phenomenally distinct *weak 1PP*, and *strong 1PP*: The weak 1PP means a purely geometric centering of the experiential space upon one’s body, and thus corresponds most to the “egocentre” (Roelofs, 1959; Merker, 2007) or “cyclopean eye” (von Helmholtz, 1962), which can be traced back to Hering’s (1942) projective geometry. Experimental work on extending the RHI paradigm has shown that the strength of illusory self-identification with a dummy or virtual body crucially depends on this kind of 1PP (Petkova and Ehrsson, 2008; Petkova et al., 2011b; Maselli and Slater, 2013), and that in addition to proprioceptive information, vestibular information is crucial for determining self-location in space (Schwabe and Blanke, 2008; Blanke, 2012).

**Figure 1:**
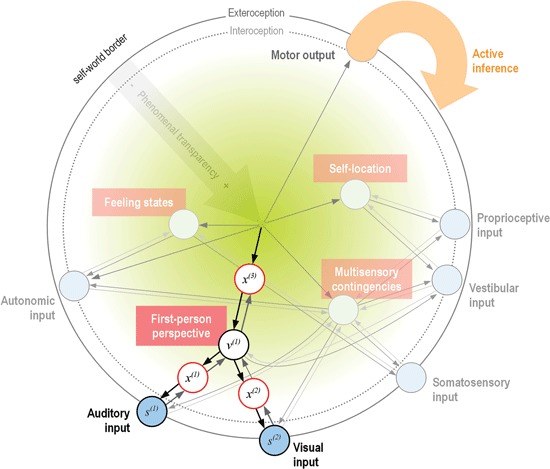
**Schematic proposal for a mapping of the *phenomenal self-model* onto a hierarchical generative model** (format adapted from Bastos et al., 2012). Shown here is only the system’s model of itself, where representational nodes at each level generate descending predictions to increasingly specialized lower levels (symbolized by darker arrows). In this special case, the single modeled cause of sensations is the *minimal phenomenal self* (Metzinger, 2004a), which generates predictions about the state of one or many sensory modalities (blue circles). The inversion of this generative model (a predictive coding scheme, lighter arrows) infers hidden causes—and thus ultimately, the self as the single cause—of sensory input via minimization of prediction error (Friston, 2011). For simplicity, only one intermediate level of nodes within the hierarchy is displayed, consisting of the basic properties of minimal selfhood as reviewed (white circles). As a (simplified) illustration of the hierarchical generative processing, the case of the 1PP is highlighted. Here, descending predictions of the unified self-model (black arrows) generate sensory data *s^(i)^* in the respective modalities (auditory and visual). This happens via a hierarchy of hidden states *x^(i)^* and hidden causes *v^(i)^* (the 1PP), which generate predictions about data in the level below. The green gradient symbolizes increasing transparency of the accompanying phenomenal states with ascending hierarchy, where the final cause (the self) is completely transparent. Note that at this (highest) level, there is no further representational node; this acknowledges the fact that the perception of a unified minimal self is the result of a temporally extended predictive process, not a static representation (Metzinger, 2004a; Hohwy, 2007). The experience of “mineness” of the self (and of perception and action in general, Hohwy, 2007) is a result of the model’s successful predictions and thus implicitly symbolized by the arrows. Input into this system-model comes from intero- and exteroception (blue circles), while active inference is a means of changing predicted input in all modalities through interaction with the environment. As the model-evidence is evidence for the agent’s existence (Friston, 2011; Friston, 2013b), the model will necessarily be a veridical model of the agent: if there was too much unexplained prediction error, the model would be abandoned in favor of a model with a higher evidence; the self in the present form would cease to exist (Hohwy, 2010; Friston, 2011; Friston, 2012b).

As an attempt to summarize the reviewed accounts of the basic constituents of MPS, Figure 1 shows a schematic depiction of a hierarchical generative model, predicting from the *minimal phenomenal self* to increasingly specific, unimodal lower levels on shorter timescales (Kiebel et al., 2008; Hohwy, 2010; Clark, 2013). For simplicity, we have only included one intermediate level in the hierarchy, consisting of the basic aspects of minimal selfhood as discussed in the reviewed articles (see Figure caption for a detailed description).

In the generative self-model (Figure 1), the first-person perspective (1PP) node should be taken as a purely geometrical point of convergence of sensory information from a particular sensory modality (a “weak 1PP”), whereas the phenomenal centeredness of the model onto the experiencing subject would correspond to a “strong 1PP” (Blanke and Metzinger, 2009). Note that although the weak 1PP and self-location usually coincide, these two phenomena can be decoupled in neurological patients with autoscopic phenomena, while MPS still seems to be normal in these conditions (Blanke and Metzinger, 2009; Blanke, 2012). This seems to speak for a probabilistic processing of minimal selfhood, and also for a relative independence of 1PP and self-location (which are therefore also modeled as separate nodes on the intermediate level of the generative model in Figure 1).

In conclusion, the experienced 1PP presents itself as a key feature of “mineness”, and thus as a basic constituent of, and a prerequisite for a minimal self (Gallagher, 2000; Vogeley and Fink, 2003; Metzinger, 2004a; Blanke and Metzinger, 2009). Some authors speak of a system’s “ability” to take the 1PP, meaning the ability to integrate and represent experience, i.e., mental states, in a common egocentric reference frame centered upon the body (Vogeley and Fink, 2003). The FEP very comfortably complies with the assumption that a body model “defines a volume within a spatial frame of reference … within which the origin of the weak 1PP is localized” (Blanke and Metzinger, 2009; Friston, 2011, 2012b). In this light, we now review the explanatory power of the FEP for mechanisms of modeling other agents.

### Modeling others

In opposition to the 1PP, the third-person perspective (3PP) is the perspective of the observer, i.e., the perspective that is taken when states are ascribed to someone else (Vogeley and Fink, 2003; Blanke and Metzinger, 2009; Fuchs, 2012). This form of perspective taking is of essential importance, for how we make sense of ourselves in a social environment depends on the representation of, and distinction between, actions and states of the self and those of others (Decety and Sommerville, 2003; Frith, 2007; Bernhardt and Singer, 2012; Farmer and Tsakiris, 2012; Frith and Frith, 2012). Traditionally, at least two distinct mechanisms have been postulated to underlie our understanding of other’s internal states: *experience sharing* and *mentalizing* (Brown and Brüne, 2012; Zaki and Ochsner, 2012). While experience sharing refers to a mere mirroring of others’ action intentions, sensations, or emotions (Gallese and Sinigaglia, 2011), the term mentalizing describes explicitly reflecting others’ internal states: in a recent review, Zaki and Ochsner (2012) define the mechanism behind mentalizing as “the ability to represent states outside of a perceiver’s ‘here and now’”, thus having both a spatial 1PP and a temporal (present versus past and future) aspect. Crucially, this involves a representation of other agents as possessing a 1PP that differs from one’s own (Farmer and Tsakiris, 2012). One can also describe these processes as simulating other PSMs (Metzinger, 2004a); in this way, a pre-reflective, phenomenally transparent self-model is necessary for the formation of higher-level cognitive and social mental concepts (Metzinger, 2003, 2004a, 2005; Edelman, 2008; Blanke and Metzinger, 2009).

Humans display first instances of experience sharing almost from birth onwards (Tomasello et al., 2005), for example, human infants as young as one hour after birth can already imitate facial gestures (Meltzoff and Moore, 1983). It hence seems that an “experiential connection” between self and others is already present in newborn infants (Gallagher and Meltzoff, 1996; Fuchs, 2012). Another example for such a pre-reflective self-other connection is sensorimotor mirroring (“neural resonance”, Zaki and Ochsner, 2012). Many studies have reported vicarious activations of the motor system by observing others’ actions (Rizzolatti and Craighero, 2004), or likewise of the somatosensory system by the observation of touch (Keysers et al., 2010) or pain to others (Bernhardt and Singer, 2012). These findings suggest a very basic, automatic activation of one’s representations to another person’s action intentions, or experience (Keysers et al., 2010; Zaki and Ochsner, 2012). There have been arguments for a link between sensory mirroring mechanisms and higher-level perspective taking abilities (see Preston and de Waal, 2002, for a discussion), suggesting that although such vicarious responses are activated automatically, they are not purely sensory-driven (Singer and Lamm, 2009).

The FEP emphasizes models of the behavior and intentions of others as a crucial determinant of our own behavior (Frith, 2007; Friston, 2012a). It has accordingly been proposed that mechanisms of social cognition are based on predictive coding as well (Baker et al., 2011; Brown and Brüne, 2012; Frith and Frith, 2012), where perspective taking can be described as forming “second order representations” (Friston, 2013b). In other words, as agents, we also have to predict the behavior of other agents, by not only generating a model of the physical world (and our body) but also of the mental world-models of our conspecifics based on their behavior (Frith, 2007; Frith and Frith, 2012). Crucially, we have to continually update our models of others’ mental states via prediction errors, because these states are not stable but vary over time (Frith and Frith, 2012). This task is far from trivial, and involves many levels of differential self-other modeling ranging from a purely spatial differentiation (other agents occupy different positions in the world) to the abstract modeling of other minds like in Theory of Mind (Vogeley and Fink, 2003; Baker et al., 2011).

Several recent accounts have proposed that associative learning updated through prediction errors is a common computational mechanism underlying both reward learning and social learning (Behrens et al., 2008; Hampton et al., 2008; Frith and Frith, 2012). Experimental evidence from these studies suggests that prediction errors code for false predictions about others’ mental states (Behrens et al., 2008; Hampton et al., 2008), and even for discrepancies between predictions of others and actual outcome of their choice (Apps et al., 2013). Interestingly, it seems that even low-level predictions can also be updated interactively. For example, dyads of individuals with similar perceptual sensitivity may benefit from interactive decision-making, as shown by an increased performance in a collective perceptual decision task during which levels of confidence were communicated (Bahrami et al., 2010). As mentioned before, if these basic predictive mechanisms fail, pathological behavior can emerge (Fletcher and Frith, 2008; Brown et al., 2013). For example, perspective taking abilities seem to be often impaired in individuals suffering from Autism Spectrum Disorder (ASD; Oberman and Ramachandran, 2007; but cf. Hamilton et al., 2007), while there is also evidence for impaired predictive coding mechanisms in ASD (Friston, 2012a).

An intriguing question is whether the brain uses the same models to generate predictions about own and other behavior. In a predictive coding account of action understanding, Kilner and colleagues (Kilner et al., 2007; Friston et al., 2011) have argued that the *mirror neuron system* is part of a generative model predicting the sensory consequences of actions, and that indeed, it seems that the brain applies the same model to predict one’s own, and others’ actions. Actions are thereby modeled on four hierarchical levels (Hamilton and Grafton, 2008): intentions, goals, kinematics, and muscles. By inversion of the model, the brain can thus infer the causes of own and others’ actions, via explaining away prediction error across these four levels. Thus the mirror neuron system is active during action observation because the “own” generative model is inverted to infer the intention underlying the observed action. A similar argument is made by Gallese and Sinigaglia (2011) (see also Goldman and de Vignemont, 2009) to explain embodied simulation in general by the fact that representations of states of the self and others’ states have the same bodily format, and thus the same constraints. Correspondingly, there is evidence that the same neuronal structures may be involved in predicting own and others’ internal states (Bernhardt and Singer, 2012), for example, in predicting how pain will feel for others (Singer et al., 2004). In sum, there is strong evidence that others’ mental states are inferred via internal models. It seems that the use of generative models by the brain can explain many of these basic, as well as more elaborated social mechanisms. Thereby (at least partially) common predictive mechanisms for self and others strongly support the notion of perspective taking as an “embodied cognitive process” (Kessler and Thomson, 2010). This is a relatively young, but promising field of research; it is up to future studies to evaluate the explanatory power of the FEP in this domain.

## Conclusion

In this review, we have summarized proposals from different authors, all emphasizing the concept of hierarchical generative models to explain processes underlying the bodily foundations of MPS, including its fundamental constituents such as multisensory integration, the sense of agency, the experience of mineness, perspectivity, and its phenomenal transparency. We have reviewed these free energy accounts of key aspects of minimal selfhood in the light of the premise that the self is the result of a generative process of self-modeling (Metzinger, 2004a; Hohwy, 2007). The approaches reviewed here show that the FEP complies with the claim that minimal selfhood emerges from physiological processes (Gallagher, 1986, 2000; Zahavi, 1999; Legrand, 2006; Blanke and Metzinger, 2009), and acknowledges both the phenomenal and spatiotemporal centeredness of the generative self-model as a key for minimal self-awareness. Albeit still schematic, these accounts demonstrate that the predictive coding account can inform theoretical and experimental approaches towards the normal and pathological self. The FEP is increasingly gaining influence as a “deeply unified account of perception, cognition, and action” (Friston, 2010; Hohwy, 2010; Apps and Tsakiris, 2013; Clark, 2013), up to recent accounts proposing it as a general mechanism underlying evolution and the “emergence of life” itself (Friston, 2013c). A particular strength of the approach seems to be that it makes relatively few conceptual assumptions (Hohwy, 2007, 2010; Friston, 2008; Friston and Kiebel, 2009; Friston et al., 2012), thus being capable of formalizing both spatial and social aspects of self-models. Of course, there are many outstanding issues, and the free energy formulation will have to withstand thorough empirical testing (for discussions, Friston et al., 2012; Apps and Tsakiris, 2013; see Clark, 2013). While it is well-established in the domains of action and perception, future work will have to show whether the FEP can be similarly influential in cognitive and social domains. Particularly, the social domain lacks models (Frith and Frith, 2012), and currently the FEP seems one of the most promising candidate theories to formally describing the mechanisms underlying the experience of being a “self in relation to others” (Frith, 2007; Friston, 2012a). The FEP may thus provide a framework to address philosophical debates about self-modeling (Gallagher, 2005b; cf. Metzinger, 2006), and perhaps help to bridge gaps between neuroscientific and philosophical approaches to the self.

## Footnotes

^1^In general, this approach is concerned with “global aspects of bodily self-consciousness” (Blanke and Metzinger, 2009), where a *global* property is something that can only be ascribed to a system as a whole, and *self-consciousness* refers to “the ability to become aware of one’s own mental and bodily states … as one’s own mental and bodily states” (Vogeley and Fink, 2003). The kind of self-consciousness meant here is not cognitive but “immediate, *pre-reflective* and non-observational” (see also Zahavi, 1999; Gallagher, 2005a; Legrand, 2006; Hohwy, 2007), where the term pre-reflective is referring to levels of self-awareness that are independent of explicit cognition and linguistic abilities (Blanke and Metzinger, 2009). In its simplest form, this is the *minimal phenomenal self*, the “fundamental conscious experience of being someone” (Blanke and Metzinger, 2009).

^2^Although the illusory experience of feeling the touch on the dummy hand is certainly surprising, one has to distinguish this cognitive surprise of the agent from “surprise” on a neurobiological level (“surprisal”, see Clark, 2013), as defined by prediction error. In fact, here these two notions may be somewhat opposing: the dummy hand is accepted as a part of one’s body as a result of successfully *explaining away* the surprise evoked by the ambiguous multisensory stimulation (Hohwy, 2010; Hohwy and Paton, 2010). However, the agent experiences exactly this state—owning a lifeless dummy hand—as surprising.

^3^But, as pointed out by Hohwy (2007, 2010), active inference is still happening at a more subtle level, as participants focus their attention on the rubber hand to detect potential mismatches of observed and felt touch.
